# The Relationship Between Perifoveal L-Cone Isolating Visual Acuity and Cone Photoreceptor Spacing—Understanding the Transition Between Healthy Aging and Early AMD

**DOI:** 10.3389/fnagi.2021.732287

**Published:** 2021-09-09

**Authors:** Rigmor C. Baraas, Åshild Horjen, Stuart J. Gilson, Hilde R. Pedersen

**Affiliations:** Faculty of Health and Social Sciences, National Centre for Optics, Vision and Eye Care, University of South-Eastern Norway, Kongsberg, Norway

**Keywords:** age-related macular degeneration, isolated L-cone acuity, cone density, aging, outer segment length, inner segment length, cone spacing

## Abstract

**Background:** Age-related macular degeneration (AMD) is a multifactorial degenerative disorder that can lead to irreversible loss of visual function, with aging being the prime risk factor. However, knowledge about the transition between healthy aging and early AMD is limited. We aimed to examine the relationship between psychophysical measures of perifoveal L-cone acuity and cone photoreceptor structure in healthy aging and early AMD.

**Methods and Results:** Thirty-nine healthy participants, 10 with early AMD and 29 healthy controls were included in the study. Multimodal high-resolution retinal images were obtained with adaptive-optics scanning-light ophthalmoscopy (AOSLO), optical-coherence tomography (OCT), and color fundus photographs. At 5 degrees retinal eccentricity, perifoveal L-cone isolating letter acuity was measured with psychophysics, cone inner segment and outer segment lengths were measured using OCT, while cone density, spacing, and mosaic regularity were measured using AOSLO. The Nyquist sampling limit of cone mosaic (N_*c*_) was calculated for each participant. Both L-cone acuity and photoreceptor inner segment length declined with age, but there was no association between cone density nor outer segment length and age. A multiple regression showed that 56% of the variation in log L-cone acuity was accounted for by N_*c*_ when age was taken into account. Six AMD participants with low risk of progression were well within confidence limits, while two with medium-to-severe risk of progression were outliers. The observable difference in cone structure between healthy aging and early AMD was a significant shortening of cone outer segments.

**Conclusion:** The results underscore the resilience of cone structure with age, with perifoveal functional changes preceding detectable changes in the cone photoreceptor mosaic. L-cone acuity is a sensitive measure for assessing age-related decline in this region. The transition between healthy aging of cone structures and changes in cone structures secondary to early AMD relates to outer segment shortening.

## Introduction

Age-related macular degeneration (AMD) is a multifactorial, chronic disease that progresses through early, intermediate, and late stages (Wong et al., 2014; NICE guideline [NG82], 2018). AMD has a very long asymptomatic phase, typically spanning more than a decade, before the disease produces symptomatic visual loss. The earliest recognizable lesion in humans *in vivo* is the deposition of drusen between the retinal pigment epithelium (RPE) and Bruch’s membrane (Mullins et al., 2000; Pedersen et al., 2018), but symptomatic visual loss may not occur before photoreceptors start to degenerate. Rods appear to be more vulnerable to aging than cones, and their function and structure degenerates earlier in AMD (Schuman et al., 2009). Cone inner segments (IS) and outer segments (OS) shorten as AMD progresses (Johnson et al., 2003), with cone IS shortening being associated with shrinking (Litts et al., 2015a) and translocation of mitochondria (Litts et al., 2015b). The neural economy hypothesis (Elsner et al., 2020) argues that cones can survive for longer because of this shortening and/or because of less photopigment allowing for a more economical configuration.

The resilience of the cones (Curcio et al., 1993) is a factor that limits our understanding of the transition between healthy retinal cone function in aging and early AMD (Curcio et al., 2020). This lack of understanding is compounded by most studies being cross-sectional in design, where the small effect size expected for a given individual is likely to be dwarfed by inter-individual variability. For example, individual differences in cone density are large, even in young, healthy eyes (Li and Roorda, 2007; Dees et al., 2011; Song et al., 2011; Zhang et al., 2015; Elsner et al., 2017; Pedersen et al., 2019). Refractive errors, such as myopia affect cone distribution (Chui et al., 2008; Wang et al., 2019) and are associated with myopic macular degeneration (Fricke et al., 2018). Another problem is the frequency of comorbidities in the elderly (e.g., Hoogendijk et al., 2016), which can affect retinal health and function in both controls and AMD participants alike. Cones, however, appear to be resilient in healthy retinal aging—any possible senescent loss of cone photoreceptors is too small to be distinguishable from the large inter-individual differences in cone density (Curcio et al., 1993; Dees et al., 2011; Song et al., 2011; Chui et al., 2012; Land et al., 2014). There is one report of shortening of cone outer and inner segments in healthy aging (Elsner et al., 2020), as well as a reported decline in mitochondrial membrane potential and cellular energy in cone IS (Eells, 2019). In parallel, it is well known that there is a senescent decline, that begins in young adulthood, in spatio-chromatic contrast sensitivity (Dees et al., 2015) and other measures of chromatic sensitivity (Knoblauch et al., 2001; Shinomori et al., 2016). Furthermore, L-cone isolating (spatio-chromatic) acuity has been reported to correlate with cone density (Baraas et al., 2017). As such, L-cone isolating acuity could reveal subtle functional changes in aging, while any corresponding loss of cones would remain difficult to quantify. Together, these findings indicate that functional changes precede structural changes in cones in healthy retinal aging, but both factors have never been assessed in the same individual nor in both healthy aging and early AMD. We hypothesized that measures of L-cone isolating acuity would depend on an individual’s cone density and deteriorate with increasing age but precede observable changes in cone density. Furthermore, because of the reported shortening of cone OS/IS in AMD (Johnson et al., 2003), the functional deterioration would be more pronounced in early AMD.

We examined the association between spatio-chromatic acuity, cone density, cone spacing, and age in the same individuals, who had either healthy eyes and retinas, or retinas with signs of early AMD. Healthy controls from young adulthood to old age were included to show the baseline senescent functional decline, and how early-AMD functional loss cannot be explained by this decline alone. The retina was imaged with optical-coherence tomography (OCT) and adaptive-optics scanning-light ophthalmoscopy (AOSLO) to assess both cone outer and inner segment length and cone density. We chose to isolate L-cone function, as L cones are typically more abundant than M cones in the retina of Caucasians (Hagen et al., 2019), and L-cone acuity (but not M-cone acuity) has been reported to correlate with cone density in healthy young adults (Baraas et al., 2017). L cones also have the type of mitochondrial activity associated with IS shortening observed in AMD (Litts et al., 2015a,b), and are the cones that appear to survive the longest in advanced AMD (Curcio et al., 1996). L-cone function is also less affected by age-related changes in the crystalline lens (Dees et al., 2015). Measurements were obtained at 5 degrees eccentricity, where inter-individual variability is less of an issue (Dees et al., 2011; Song et al., 2011; Zhang et al., 2015). This would also circumvent factors that may make the fovea less sensitive to functional changes, such as the redundancy of cones in the foveal center (Dees et al., 2011; Bensinger et al., 2019), and that the central foveal cones are somewhat protected by Müller glia cells and macular pigment (Curcio et al., 2020).

## Materials and Methods

### Participants and Baseline Measurements

In total, 39 males and females, all Caucasian background, were included in this cross-sectional study. Ten individuals (aged 61–78 years), exhibiting signs of early AMD as classified by NICE NG82 (2018), were included in the early AMD group. Twenty-nine were included in the healthy control group (aged 15–70 years). The broad age range in the control group was necessary to measure the expected baseline senescent decline in spatio-chromatic acuity (Dees et al., 2015); to observe that spatio-chromatic acuity correlates with cone density (Baraas et al., 2017) across the large inter-individual variation in cone density (Dees et al., 2011; Song et al., 2011; Zhang et al., 2015; Elsner et al., 2017); and that cone density was expected to be minimally affected by age (Curcio et al., 1993; Dees et al., 2011; Song et al., 2011; Chui et al., 2012; Land et al., 2014).

All participants were healthy with no known ocular pathology (other than AMD for those in the AMD group), and no former intraocular or refractive surgery and/or systemic diseases. All had corrected-to-normal logMAR visual acuity (early AMD group: −0.14 to 0.3; control group: −0.20 to 0.0, TestChart 2000, Thomson Software Solutions, London, United Kingdom). Ocular pathology was assessed with slit-lamp biomicroscopy, fovea-centered digital 45-degrees color fundus photographs (Topcon TRC-NW6S non-mydriatic fundus camera, Topcon Corp., Tokyo, Japan) and high-resolution OCT (Heidelberg Spectralis OCT2, Heidelberg Engineering GmbH, Germany). Grading of small hard drusen was performed as described previously (Pedersen et al., 2018). Axial length, corneal curvature, anterior chamber depth, and central corneal thickness were measured with the IOLMaster 700 (Carl Zeiss Meditec AG, Jena, Germany). All had normal color vision except one (5503) who had a known deutan deficiency, as screened with the Ishihara (24 pl. ed., Kanehara Trading INC, Tokyo, Japan, printed 2005) and the Hardy-Rand-Rittler 4th edition (Richmond Products, Albuquerque, NM) tests. All tests were performed following standard procedures. The initial assessment took about one hour for each participant.

### Ethics Statement

The study was approved by the Regional Committee for Medical Research Ethics for the Southern Norway Regional Health Authority and was carried out in accordance with the principles in the Declaration of Helsinki. Informed consent was obtained from all the participants included in the study after full explanation of the study procedures.

### OCT Imaging

**FIGURE 1:**
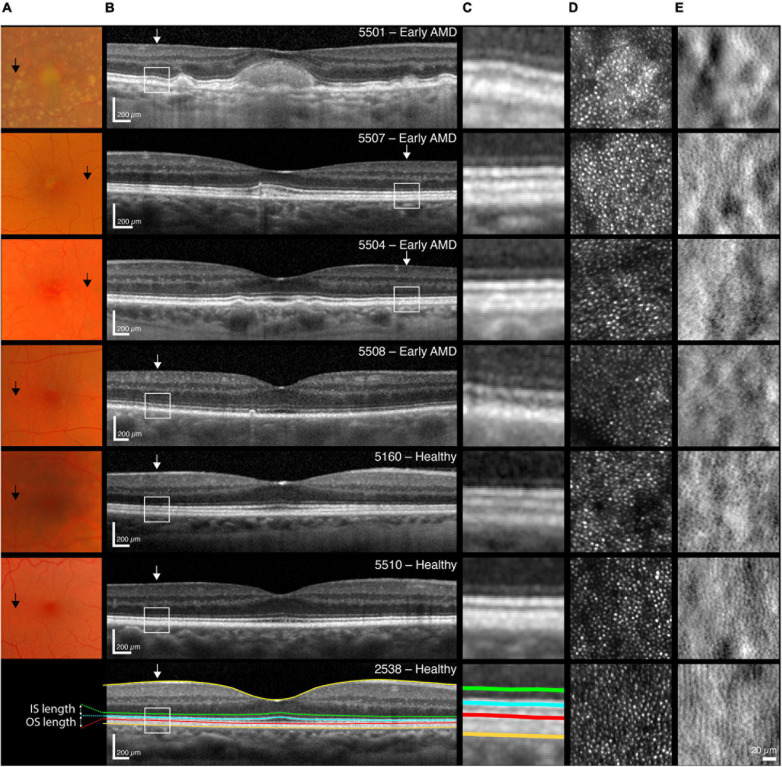
Multimodal images of one participant with early AMD and high-risk of progression (5501, top row), followed by one with medium risk (5507) and two with low-risk (5504, 5508); as well as three normal controls [5160 (68 years), 5510 (65 years), 2538 (35 years)]. **(A)** Color fundus photos of the central 14 degree of the macula. Black arrows indicate 5 degree temporal eccentricity. **(B)** Horizontal SD-OCT scan through the foveal center of the central ±7 degree including an illustration of the segmented inner limiting membrane (ILM, yellow line), the external limiting membrane (ELM, green line), the inner/outer segment junction also referred to as the inner segment ellipsoid zone (EZ, blue line), the cone outer segment tips also referred to as the interdigitation zone (IZ, red line) and the retinal pigment epithelium—Bruch’s membrane layer (RPE-BrM, orange line). The inner segment (distance from ELM to EZ) and outer segment (distance from EZ to IZ) lengths were extracted and used for analyses. White arrows indicate 5 degree temporal eccentricity. Scale bars = 200 μm. **(C)** A magnification of a one-degree area centered at 5 degree eccentricity, delineated by white boxes in B. Images from 5501 had visible drusen in this area and showed marked thickening in the RPE layer. None of the other participants with early AMD showed any apparent retinal changes within this area. **(D)** Raw confocal and **(E)** split-detection images showing reflection from cone outer segments and multiple-scatter image of cone inner segments at 5 degree eccentricity (temporal). Images are 150 × 150 μm or approximately 0.52 × 0.52 degree, scale bar = 20 μm.

High-resolution OCT images were acquired with the Heidelberg Spectralis OCT2 (30 × 5 degrees volume; 49 horizontal B-scans; 1536 A-scans per B-scan; 20 frames averaged). The registered and averaged OCT images were scaled for each participant’s individual retinal magnification ratio using the Gullstrand four-surface schematic eye model (Wang et al., 2019). The retinal layers were segmented using a semi-automatic active contour method, as described previously (Pedersen et al., 2020). After segmenting the inner boundary of the inner limiting membrane (ILM), successive layers were then segmented at the center of the external limiting membrane (ELM), ellipsoid zone (EZ), and interdigitation zone (IZ), and the posterior boundary of the RPE-Bruch’s Membrane (RPE-BrM) band. The foveal center was defined as the section with maximum outer segment length (EZ to IZ) and minimum foveal thickness (ILM to RPE-BrM) within the foveal pit. The B-scan that passed through the defined foveal center was used for analysis. Retinal thickness, outer segment (OS) and inner segment (IS) length measures were extracted over a 0.5 degree region centered on 5 degrees eccentricity (for details see Figures 1B,C).

### Adaptive Optics Scanning Light Ophthalmoscope Imaging

High-resolution confocal images were acquired with the Kongsberg AOSLO instrument using the 790-nm light channel (Pedersen et al., 2018). The participant’s pupil was dilated, and accommodation suspended by instillation of cyclopentolate 1% (those aged < 30 years) or Tropicamide 0.5% eye drops prior to imaging. A dental impression on a bite bar stabilized the head and provided stable pupil positioning during imaging. The macular region was imaged from 0–6 degrees eccentricity along the horizontal meridian. Images were acquired simultaneously using 1 × 1 degree fields of view and were processed according to previously published methods (Li and Roorda, 2007; Dubra and Harvey, 2010; Cooper et al., 2016). The registered and averaged AOSLO images were scaled for each participant’s retinal magnification ratio in the same way as the OCT images. The processed images were stitched together into a mosaic aligned to the corresponding infrared en-face image acquired simultaneously with the OCT B-scans (Pedersen et al., 2019). The foveal center was identified anatomically on images as described previously (Pedersen et al., 2019). Individual cones were identified via a semi-automatic algorithm (Li and Roorda, 2007; Garrioch et al., 2012). Manual cone selections were made when some were too dim to be detected automatically, based on the assumption that foveal cones are packed into a nearly hexagonal mosaic, using the non-confocal (split detector) images to disambiguate cones from rods. After manual editing, inter- and intra-cell statistics were obtained from the Voronoi tessellation of these cells including, notably, the mean number of neighbors and the mean inter-cell distance (ICD). ICD was used to calculate the Nyquist sampling limit of the cone photoreceptor mosaic (N_*c*_), where $N_{c}=\frac{\sqrt{3}}{2}\times ICD$. Cone density was estimated over six 50 × 50 μm sampling windows at 5 degrees temporal eccentricity. The percentage of 5-, 6-, or 7-sided Voronoi cell neighbors was calculated to characterize the regularity of the photoreceptor mosaic.

### Perifoveal L-Cone Isolating Single Letter Visual Acuity

Eight in the AMD group (aged 61–78) and fourteen of the healthy controls (aged 24–70 years) also performed the psychophysical tests (L-cone acuity). L-cone isolated spatial acuity was measured with a tumbling Sloan E letter of 23% increment cone contrast as described elsewhere (Baraas et al., 2017). Briefly, the background was always 10 cd/m^2^ with chromaticity metameric to CIE illuminant D65 and the CIE (x, y, Y)-coordinate of the L-cone isolating Sloan E stimulus at 23% was (0.385, 0.316, 11.5). The stimuli were displayed on a calibrated 22-inch CRT monitor (ViewSonic P227f, ViewSonic Corporation, Walnut, CA, United States). The participants were placed comfortably in a chin- and forehead-rest and viewed the display monocularly, with their preferred eye, using appropriate refractive correction for the given viewing distance of 229.2 cm. The experiment was carried out in an otherwise darkened room. Prior to each experiment, the participants were dark adapted for 3 min, then light adapted for 1 min by viewing a neutral gray screen with the same color and luminance as the background of the stimuli. A four-alternative forced-choice procedure was implemented, and the size of the Sloan E was altered using an adaptive procedure (Kontsevich and Tyler, 1999; Prins and Kingdom, 2018), and analyzed as described previously (Baraas et al., 2017). The participant’s task was to maintain fixation on a black cross that appeared at the side of the display, while the Sloan E was presented at 5 degrees temporal eccentricity for 250 ms. The participant indicated whether the Sloan E was oriented up, down, left, or right by pushing the corresponding response button. Failure to maintain fixation was automatically detected by the software and the trial re-tested with a different orientation of the Sloan E. Fixation was verified with an eye tracker, which monitored direction of gaze 120 times per second (ASL Eye Tracking System Model 5000, Applied Science Laboratories, Bedford, MA, United States).

### Data Analysis

All data analyses were performed with the statistical software R, version 4.0.5 (R Core Team, 2021). Mean, standard deviation, median, range, and Z-scores were calculated for descriptive analysis.

Welch’s 2-sample *t*-test (adjusts degrees of freedom to account for variance differences between the samples) were used for comparisons between participants with early AMD and healthy controls, as the data approached a normal distribution (as assessed by QQ-plots, Shapiro-Wilk test, and histograms). The homogeneity of the variances across groups was assessed using the F-test. Correlations were assessed using Pearson (r) coefficients. Multiple linear regressions were performed to assess the relationship between log N_*c*_ and L-cone logMAR at 5 degrees eccentricity with age as a covariate. Significance level was set at 0.05. For retinas exhibiting signs of early AMD, cones at 5 degrees eccentricity were semi-automatically identified twice, 6 weeks apart, by one experienced grader (author HRP). The intraclass correlation coefficient (ICC) were calculated for 50 pairs of perifoveal cone density measurements using the **irr** package (version 0.84.1) in R.

## Results

### Clinical Characteristics

Tables 1, 2 show the clinical characteristics of all AMD participants and healthy controls, respectively. Presence of drusen and their size and texture were assessed within 2 disk diameters of fovea. Of those with AMD, three had metamorphopsia (5500, 5501, and 5503) when assessed with Amsler chart. None had lens nuclear opacity more than NO 2 according to LOCS III (Chylack et al., 1993). None had cortical or posterior subcapsular opacities. All the AMD participants had early AMD, 6 with low risk and 4 with medium-to-high risk of progression according to NICE NG82. The grading encompasses the observed changes in the fovea in these participants (see Supplementary Material for OCT B-scans with a more detailed description than that provided in Table 1). A qualitative assessment of the fundus and OCT images showed no apparent retinal changes around 5 degrees eccentricity in any of the AMD participants, except participant 5501 who had small and medium drusen in this area (see Figure 1, top row). Five of the AMD participants had a disrupted inner segment ellipsoid zone (EZ) (also called inner/outer segmented junction) over one or more drusen at other macular locations. Those with AMD were 61 years or older, whereas eight healthy controls were 50 years or older. None of the controls had metamorphopsia when tested with Amsler chart, nor any gradable lens opacities. None of the healthy controls had any sign of retinal diseases, although eight had 1–3 small hard drusen which are normal age-related changes. One participant with a known color deficiency (5503) failed both pseudoisochromatic plate tests, while the others—in both the AMD and the control group—made 2 or fewer errors, as would be expected of normal trichromats. All participants demonstrated stable central fixation on both OCT and AOSLO.

**TABLE 1:** Clinical characteristics of the 10 participants with early AMD presenting age, sex, eye tested, spherical equivalent refractive error (SER), ocular axial length (AL), logMAR visual acuity, frequency of drusen, drusen size and type, the presence of disrupted ellipsoid zone (EZ), pigmentary changes, and clinical classification of AMD according to NICE NG 82 (NICE guideline [NG82], 2018).

**ID**	**Age**	**Sex**	**Eye tested**	**SER**	**AL**	**VA**	**# of drusen**	**Size** *	**Type**	**Disrupted EZ**	**Pigment changes**	**OS length**	**IS length**	**Classification**
5508	61	M	OD	−1.25	26.33	−0.14	15–20	S–M	Hard	Yes	No	21.11	29.01	Early: low risk
5506	72	M	OS	+1.00	23.34	−0.10	20–25	S–M	Both	Yes	No	24.46	27.07	Early: low risk
5503^§^	77	M	OD	+0.10	23.64	0.10	10–15	S–M	Hard	No	No	24.35	24.09	Early: low risk
5502	78	F	OD	+1.41	23.52	0.16	5–10	S–M	Both	No	No	24.98	24.81	Early: low risk
5204	71	F	OS	−0.13	24.24	−0.10	5–10	M	Soft	No	No	22.73	19.35	Early: low risk
5504	61	M	OS	−1.53	24.41	−0.10	15–20	M	Both	No	No	20.85	28.12	Early: low risk
5505	67	F	OS	−4.50	26.05	0.10	25–30	S–L	Both	Yes	No	22.25	23.70	Early: medium risk
5500^§^	66	M	OS	−1.13	24.49	0.10	5–10	S–M	Soft	Yes	Yes	19.75	25.91	Early: medium risk
5507	68	F	OS	+3.00	21.29	0.02	5–10	M	Soft	No	Yes	22.29	27.29	Early: medium risk
5501^§^	77	M	OD	+3.32	23.13	0.30	55–60	M–L	Both	Yes	Yes	21.41	23.51	Early: high risk
**Mean (SD)**	**70 (6)**			**0.03** **(2.32)**	**24.04** **(1.45)**	**0.03** **(0.14)**						**22.42** **(1.73)**	**25.29** **(2.83)**	

*Participants are sorted according to AMD severity (rightmost column).*

*S, Small drusen ≤63 μm; M, medium drusen >63 μm and ≤125 μm; L, large drusen >125 μm.

*^§^Metamorphopsia when tested with Amsler chart. Mean (SD) are in bold.*

**TABLE 2:** As Table 1 but for the 29 normal controls.

**ID**	**Age**	**Sex**	**Eye tested**	**SER**	**AL**	**VA**	**# of drusen**	Size*	**Type**	**OS length**	**IS length**	**Classification**
5181	15	F	OS	+1.67	22.44	−0.10	0			22.09	28.61	No changes
5188	15	F	OD	+0.83	23.98	−0.10	0			22.27	30.97	No changes
5159	16	F	OS	+5.18	20.63	0.02	0			17.47	29.23	No changes
5170	20	M	OD	+0.38	24.07	−0.16	0			23.76	27.78	No changes
5171	20	M	OD	−2.70	24.98	−0.20	0			24.06	27.69	No changes
5007	21	F	OS	−2.50	25.10	0.00	2	S	Hard	24.23	26.27	Normal changes
5166	21	F	OD	−2.05	24.76	−0.14	0			21.79	28.30	No changes
5169	21	F	OD	+0.19	23.53	−0.14	0			21.04	30.62	No changes
5176	21	F	OD	+0.12	22.67	−0.22	0			25.55	31.55	No changes
8323	22	F	OD	−2.70	24.95	−0.10	0			23.46	26.37	No changes
8340	24	F	OD	+0.17	22.86	−0.06	0			30.89	29.08	No changes
4017	28	M	OS	+0.88	24.01	−0.10	1			23.22	25.16	No changes
5194	33	F	OS	+1.21	22.90	−0.10	0			25.25	27.98	No changes
5197	34	F	OD	+0.45	24.03	−0.10	1	S	Hard	23.48	27.15	Normal changes
2538	35	M	OD	−4.25	24.20	−0.18	0			23.95	26.35	No changes
5165	35	F	OD	+0.12	21.63	0.00	0			24.56	26.89	No changes
4078	37	F	OD	−3.50	23.36	−0.18	0			23.88	30.31	No changes
4064	45	M	OD	+0.75	23.70	−0.10	1	S	Hard	22.46	30.33	Normal changes
4571	47	F	OS	+1.38	22.14	−0.20	0			26.64	26.69	No changes
5196	48	F	OD	−0.08	23.32	−0.10	3	S	Hard	27.87	22.75	Normal aging changes
4027	49	F	OD	+0.10	24.13	−0.10	1	S	Hard	23.21	24.84	Normal aging changes
5205	50	M	OD	−1.68	24.55	−0.08	0			27.08	27.97	No changes
5156	53	F	OD	+1.21	22.20	0.00	2	S	Hard	22.27	25.14	Normal changes
5163	56	M	OD	+0.13	23.45	−0.08	0			28.66	26.04	No changes
4049	60	M	OD	−0.63	24.26	−0.08	0			23.39	25.23	No changes
5510	65	F	OD	0.50	22.59	−0.10	2	S	Hard	26.67	25.51	Normal changes
5160	68	M	OD	−0.50	23.98	−0.08	1	S	Hard	21.69	24.60	Normal changes
5509	69	F	OS	2.25	22.89	−0.20	0			27.02	26.36	No changes
5185	70	M	OD	0.75	23.80	−0.10	0			24.89	28.70	No changes
**Mean (SD)**	**38 (18)**			**−0.08** **(1.90)**	**23.49** **(1.06)**	**−0.11** **(0.06)**				**24.23** **(2.65)**	**27.40** **(2.15)**	

*None of the normal controls had disrupted ellipsoide zone (EZ) or any pigmentary changes related to clinical classification of AMD according to NICE NG 82. Participants are sorted according to age. Mean (SD) are in bold.*

### IS/OS Thickness Measures From OCT Imaging

Figures 1A,B shows fundus images and horizontal SD-OCT scan through the foveal center of the central ±7 degrees for four representative participants with early AMD and three age-matched healthy controls. OCT images with sufficient quality for analysis were obtained from 10 with AMD and 29 healthy controls. There was a significant correlation between age and photoreceptor inner segment length, but not between age and photoreceptor outer segment length at 5 degrees eccentricity [for healthy controls only *r* = −0.51 (95% CI: −0.74 to −0.18), *p* = 0.005 and *r* = 0.34 (−0.03 to 0.63), *p* = 0.071, respectively, *n* = 29; for all *r* = −0.57 (−0.75 to −0.31), *p* = 0.0002 and *r* = 0.043 (−0.50 to 0.12), *p* = 0.79, respectively, *n* = 39). There was a significant difference in OS length at 5 degrees eccentricity between early AMD and healthy controls aged 50 years and older [−2.8 (−5.08 to −0.49) μm, *t*_(11.9)_ = −2.65, *p* = 0.0213]. There was no significant difference in IS length between early AMD and healthy controls aged 50 years and older [−0.90 (−3.12 to 1.30) μm, *t*_(13.9)_ = −0.88, *p* = 0.39].

### Cone Density and Spacing From AOSLO Cone Photoreceptor Imaging

Figures 1D,E shows multimodal AOSLO images of cones at 5 degrees temporal eccentricity for four representative participants with early AMD and three healthy controls (two of whom were age-matched with the AMD group). AOSLO images with sufficient quality for analysis were obtained from 9 with AMD and 28 healthy controls. The mean ± SD (full range) difference in perifoveal cone counts for the AMD data set was 130 ± 719 (−1,278 to 1,797) cones/mm^2^, and excellent intra-grader repeatability was observed, ICC (95% CI) 0.967 (0.942–0.981). Table 3, right part, shows the participants’ cone density and inter-cone distance (ICD) measurements at 5 degrees temporal eccentricity. Cone spacing is also presented as Nyquist sampling limit (N_*c*_) based on ICD. There was no correlation between age and cone density [for healthy controls only *r* = −0.16 (95% CI. −0.50 to 0.23), *p* = 0.43, *n* = 28; for all *r* = −0.21 (−0.50 to 0.12), *p* = 0.21, *n* = 37]. There was no difference in cone density between the groups [−1,312 (−3,289 to 656) cones/mm^2^, *t*_(13.1)_ = −1.43, *p* = 0.17], with all participants with AMD (12,829–20,473 cones/mm^2^) having cone density within the normal range (12,248–22,163 cones/mm^2^) at 5 degrees eccentricity. In terms of cone mosaic regularity, the mean ± SD percentage of cells with six neighbors was 48.6 ± 4.6% and 53.1 ± 5.8%, for the AMD group and control group, respectively, while the mean percentage of cells with five-, six-, or seven-neighbors was 94.9 ± 2.0% and 96.9 ± 1.9%. The AMD group had significantly lower percentage of cones with 6 neighbors and 5–7 neighbors than the healthy controls at 5 degrees eccentricity [−6.5 (−8.4 to −0.5)%, *t*_(17)_ = −2.39, *p* = 0.029 and −2.0 (−3.6 to 0.4)%, *t*_(12.9)_ = −2.72, *p* = 0.0176, respectively].

**TABLE 3:** Results from perifoveal L-cone isolated Sloan-E letter acuity measured, cone photoreceptor imaging, inter-cone distance (ICD), and Nyquist sampling limit (N_*c*_) at 5 degree eccentricity.

**ID**	**Age**	**L-cone Sloan-E (logMAR)**	**Linear cone density (cones/mm^2^)**	**ICD (μm)**	**N_*c*_ (arcmin)**
5508	61	0.67	15,142	9.02	1.45
5506	72	0.72	18,066	8.23	1.51
5503	77	0.71	20,473	7.77	1.43
5502	78	0.84	NA	NA	NA
5204	71	0.75	14,828	9.03	1.62
5504	61	0.66	15,180	8.98	1.56
5505	67	0.87	14,215	9.24	1.48
5500	66	NA	12,829	9.73	1.67
5507	68	NA	18,293	8.18	1.70
5501	77	0.90	17,243	8.41	1.58
Mean (SD)	69.8 (6.3)	0.77 (0.09)	16,252 (2,412)	8.73 (0.62)	1.56 (0.10)
8340	24	0.62	17,387	8.35	1.60
4017	28	0.59	18,498	8.11	1.46
5194	33	0.67	16,138	8.69	1.66
2538	35	0.69	16,056	8.70	1.51
5165	35	0.71	18,718	8.06	1.63
4078	37	0.51	20,228	7.79	1.41
4064	45	0.54	20,179	7.78	1.40
4571	47	0.65	16,489	8.58	1.70
4027	49	0.67	14,484	9.18	1.64
5163	56	0.70	20,239	7.72	1.42
4049	60	0.72	16,505	8.60	1.52
5510	65	0.76	19,711	7.86	1.55
5160	68	0.65	18,613	8.08	1.44
5509	69	NA	16,841	8.54	1.51
5185	70	0.74	16,326	8.64	1.56
Mean (SD)	48.1 (15.8)	0.66 (0.07)	17,761 (1,824)	8.31 (0.43)	1.53 (0.10)

*AMD participants (top 10 rows) are sorted according to AMD severity (see Table 1), whereas healthy controls (bottom 15 rows) are sorted according to age.*

### Perifoveal L-Cone Isolating Single Letter Acuity From Psychophysics

**FIGURE 2:**
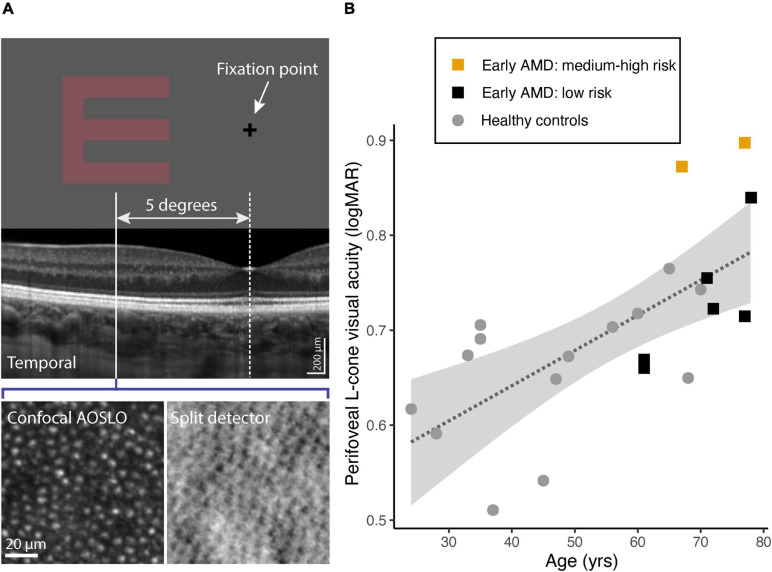
**(A)** The L-cone isolating Sloan E presented at 5 degree temporal eccentricity. SD-OCT, confocal and spit-detection AOSLO images were analyzed at the corresponding retinal location. **(B)** L-cone logMAR as a function of age: 14 healthy controls (gray filled circles) and 8 participants with early AMD: 6 with low risk of progression (black filled squares) and 2 with medium or high risk of progression (orange filled squares). The dashed line shows the best-fit linear regression to the data. The gray shaded area indicates the 95% confidence intervals for the fitted regression line.

Figure 2A shows an illustration of the Sloan-E stimulus used for measuring L-cone isolating acuity at 5 degrees eccentricity. Table 3, middle part, shows the results from measures of perifoveal L-cone isolating Sloan-E acuity for those who performed the test, 8 with early AMD and 14 healthy controls. One elderly healthy control and two with AMD did not complete the experiment because of problems with handling the response box. The participant with a deutan color deficiency (5503) was included, because their deficiency mainly affects the M-cone photopigment, not L-cone. Figure 2B shows L-cone acuity as a function of age for these 22 participants. L-cone acuity was poorer with increasing age [*r* = 0.679 (95% CI: 0.36–0.86), *p* = 0.0005, *n* = 22]. There was a significant difference between healthy controls aged under- and over-50 years [*t*_(11.6)_ = 2.93, *p* = 0.013], with the older age group having poorer L-cone acuity (mean ± SD 0.716 ± 0.04) than the younger age group (0.628 ± 0.07). Perifoveal L-cone acuity did not correlate with axial length or spherical refractive error, neither for AMD nor for healthy controls. The difference in perifoveal L-cone acuity between all eight participants with AMD and healthy controls aged 50 years and older was not significant [0.05 (−0.03 to 0.13), *t*_(10.6)_ = 1.33, *p* = 0.21]. Nevertheless, the two participants who had the most severe form of early AMD (5501, 5505) had poorer L-cone acuity than that expected from age alone, with values outside the 95% confidence interval of the regression line (Figure 2B).

### Relationship Between L-Cone logMAR, log N_*c*_, and Age

**FIGURE 3:**
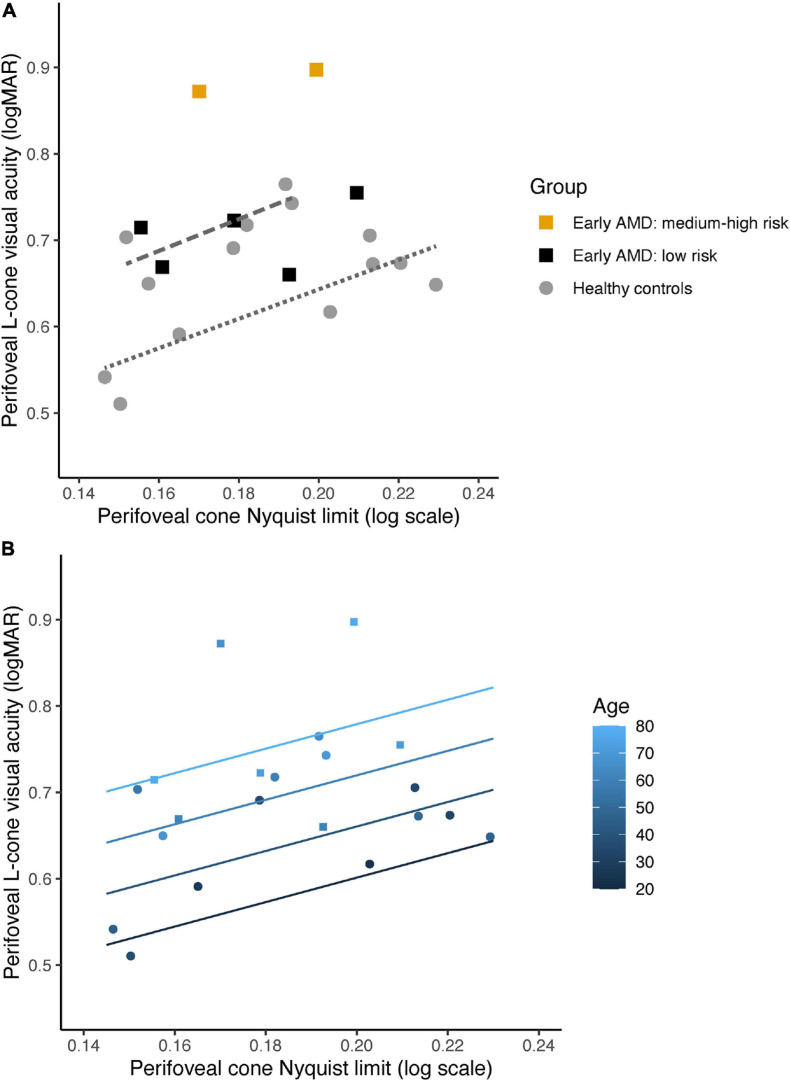
**(A)** Perifoveal L-cone visual acuity as a function of cone Nyquist sampling limit (N_*c*_) for 14 healthy controls (gray filled circles) and 7 participants with early AMD: 6 with low risk of progression (black filled squares) and 2 with medium or high risk of progression (orange filled squares). Note that data from 5502 is not included in this figure, as we did not obtain AOSLO images of sufficient quality for analysis. Regression lines for log N_*c*_ and L-cone acuity for the healthy controls aged 50 years and older (dashed line) and younger than 50 years (dotted line), respectively. **(B)** Predicted regression models for perifoveal L-cone visual acuity as a function of N_*c*_ at 20, 40, 60, and 80 years of age for healthy controls (filled circles), color coded from dark blue (youngest) to light blue (oldest). Data for the participants with early AMD are also shown (filled squares).

Figure 3A show a linear relationship between perifoveal log L-cone visual acuity and log cone Nyquist sampling limit (N_*c*_). The data for the healthy controls are best explained by fitting two regression lines, one each for those aged younger or older than 50 years, respectively [*R*^2^ = 0.72, *F*_(2, 11)_ = 17.33, *p* = 0.0004]. The acuities of those with early AMD and low risk of progression overlap with the healthy controls. However, the two with early AMD and medium-to-high risk of progression (5501, 5505) had considerably poorer L-cone acuity than that expected based on their N_*c*_. Because L-cone logMAR is associated with N_*c*_ as well as with age, a multiple regression was performed to assess if perifoveal log L-cone acuity could be predicted from log N_*c*_ with age as a covariate, both for the healthy controls alone and for controls together with early AMD participants. A significant regression was found with log N_*c*_ predicting L-cone acuity both for the healthy controls [*R*^2^ = 0.57, *F*_(2, 11)_ = 7.171, *p* = 0.01, *n* = 14] and for controls and early AMD [*R*^2^ = 0.56, *F*_(2, 18)_ = 11.48, *p* = 0.0006, *n* = 22] when taking age into account. A linear model in which group (control vs. AMD) was included as a separate factor did not fit the data better than one in which no group variable was included [Likelihood Ratio Test: *F*_(1, 17)_ = 0.514, *p* = 0.48]. The two participants (5501, 5505) who had poorer L-cone acuity than expected from their Nyquist sampling limit and age, when considered separately, appeared as outliers in the diagnostic plots. Fitting the data with these two participants excluded had no effect on the significance tests nor on the interpretation of the results. The predicted regression models based on healthy controls are visualized in Figure 3B, which show a parallel shift in N_*c*_-dependent L-cone acuity with healthy aging.

## Discussion

We have shown that there is an age-related decline in perifoveal L-cone function, a decline that begins in young adulthood without any observable changes in cone density and spacing, supporting the main hypothesis. The decline in L-cone function was greater in those with early AMD and preceded any changes in cone density or spacing. This functional decline parallels inner segment shortening in healthy eyes, whilst it parallels inner and outer segment shortening in early AMD. This was the case in those with low or medium risk of progression even in areas without visible changes in RPE/Bruch’s membrane. There was no age-related change in photoreceptor OS length in healthy controls, as previously reported (Elsner et al., 2020). In addition, photopigment optical density in OS of perifoveal L and M cones has been reported to increase, not decrease, with age (Renner et al., 2004). As the loss of function in healthy controls cannot be explained by loss of cones or changes of OS length, the most parsimonious explanation is that it is related to IS shortening. Thus, it may be that the combined effect of normal age-related IS shortening and the OS shortening associated with early AMD together explain the significant loss of L-cone function observed in those with early AMD and medium-to-high risk of progression. This indicates that the transition between healthy aging and early AMD appears to be mainly related to structural changes in outer segment length at 5 degrees eccentricity.

There was no age-related change and no difference between early AMD and healthy controls in cone density and spacing at 5 degrees eccentricity. This is consistent with that reported from histology (Curcio et al., 1993, 1996) and *in vivo* imaging (Song et al., 2011; Chui et al., 2012; Land et al., 2014). There are reports of changes in cone density and spacing over small hard drusen in healthy adults (Pedersen et al., 2018), and in more severe forms of early AMD (Land et al., 2014). Here, only mosaic regularity differences indicated structural changes related to cone photoreceptors in early AMD, possibly because the areas assessed had no visible changes in RPE/Bruch’s membrane. The observed regularity disruptions in those with early AMD are likely related to rod degeneration as this is known to precede any cone degeneration in AMD (Curcio et al., 1996). When rods die off during healthy aging, the space is filled in by larger rod inner segments leaving the mosaic intact (Curcio et al., 1993).

In a normal human trichromat, L cones are typically twice as numerous as M cones (with S cones making up less than 10% of the total number) and so cone spacing alone would not be expected to fully explain L-cone acuity. Both cone spacing (expressed as Nyquist sampling limit) and age, were significant predictors of log L-cone acuity measured at 5 degrees eccentricity, but cone spacing alone did not correlate with L-cone acuity in early AMD. Note that 5505, who also has an abnormally long axial length and is myopic, has a Nyquist sampling limit that lies roughly in the middle of the distribution (Figure 3A, left most orange filled square) but has an L-cone acuity that is still poorer than that expected from her age alone. Healthy controls older than 50 have poorer L-cone acuity but retain a Nyquist sampling limit within the same range as those younger than 50. Similarly, the regression models imply (Figure 3B) that function, even in healthy aging, deteriorates with age while cone spacing appears to remain stable. Both the observed photoreceptor IS shortening with healthy aging, and OS shortening in AMD without any evidence of age-related changes in number of cones, are in line with the neural economy hypothesis—that cones adapt to survive in “harsh conditions” (Elsner et al., 2020). The photoreceptor IS shortening at 5 degrees eccentricity reported here relates to shortening of both rod and cone inner segments and appears to be a normal aging process, perhaps to uphold some degree of functionality.

Photoreceptor inner segments are rich in mitochondria (Hoang et al., 2002), and mitochondrial function, in general, declines with aging as well as contributing to an increase in the generation of reactive oxygen species (Lopez-Otin et al., 2013). Alterations to the IZ band and IS shortening within 3–4 degrees of eccentricity have previously been reported to be associated with decreased cone function in patients with early AMD (Wu et al., 2013) and with mitochondrial dysfunction (Fritsche et al., 2008). It is reasonable to attribute loss of L-cone function with decline in mitochondrial function, as mitochondria contribute to IS optical properties (Hoang et al., 2002). However, it cannot be ascertained whether the observed decline in photoreceptor IS length in early AMD can be attributed to normal age-related mitochondrial changes only, before cone OS degenerates in later stages of AMD (Mitamura et al., 2013). Degeneration of cone OS in AMD has been reported to contribute to further shrinking and translocation of mitochondria (Litts et al., 2015a,b). Previous studies have also shown that OS are the first to be affected in both degenerating rods and cones in areas over drusen (Zhang et al., 2014). The results here show that there are observable OS changes in early AMD even in areas where there are no visible changes in the RPE/Bruch’s membrane (Figure 1). This adds to the body of evidence that AMD is a global retinal phenomenon, not a local one. From OCT images, however, we cannot disentangle the differences between rods and cones, except that the ratio of rods to cones within the perifovea is reported to be 9:1 in younger vs. 6:1 in older adults (Curcio and Allen, 1990).

### Strengths and Limitations

A major strength of this study was that it was the first to examine both isolated L-cone functional and photoreceptor structural measures in the same individuals, both in healthy aging and early AMD. Such cross-sectional measures of structure and function may be considered a limitation because they do not have single cell resolution (Duncan and Roorda, 2019), but the robustness of the results indicates that they may have larger clinical utility. Perifoveal L-cone acuity is, potentially, an easy-to-use clinical measure. Our data provides new motivation for investigating sensitive functional measures combined with multimodal imaging to advance the understanding of the transition between healthy aging and early AMD. As seen from OCT images, the differences in OS length and associated vision loss between healthy aging and early AMD in structurally intact parts of the retina may be a very sensitive indicator for risk of progression and potentially a way to monitor treatment success. Measures of isolated L-cone acuity may be particularly useful for measuring cone function, as it is mainly red and green cones that survive without OS in advanced AMD (Curcio et al., 1996).

A limitation of this study was the small number of participants with early AMD and few age-matched controls. The study only included Caucasians, and from an age range that did not include anyone older than 70 (healthy controls) and 78 (early AMD) years old, potentially limiting the generalizability of our results. Despite this limitation, the observed continuous age-related decline in L-cone acuity from young adulthood is the same as that observed for other measures of chromatic sensitivity (Knoblauch et al., 2001; Dees et al., 2015; Shinomori et al., 2016). Furthermore, the inclusion of the more elderly low-risk AMD participants helps to emphasize the differences from those with the more severe form of early AMD.

For the L-cone acuity measures, a technical limitation arose that the response-box buttons were too small for three of the elderly participants, preventing them from reliably providing the correct response within the time limit. For the AOSLO instrument, the eldest participant was unable to maintain a stable tear film, which prevented us from acquiring usable images. A limitation of the OCT imaging was the inability to distinguish rods from cones, in terms of OS and IS lengths, at 5 degrees eccentricity. Additionally, the IZ sometimes appeared diminished and indistinguishable from the reflection from the RPE (see for example 5508 in Figure 1B). In these cases, the IZ was segmented at the inner boundary of the RPE reflection, which could overestimate the OS length measurement slightly. Even with this possible over-estimation, OS length in early AMD participants remained shorter than those in controls.

## Conclusion

We demonstrate that perifoveal L-cone acuity is a sensitive measure for assessing age-related decline in cone function and is more readily detectable than cone photoreceptor mosaic changes. This lends strong support to the neural economy hypothesis (Elsner et al., 2020). The results indicate that the transition between healthy aging of cone structures and changes due to early AMD relates to OS shortening, but more data is needed to understand to what degree OS and IS shortening contribute to the observed decline in L-cone function in early AMD. Vulnerability to loss of cone function secondary to early AMD may very well depend on the cone-richness of the individual’s macula. In addition, a healthy lifestyle may delay normal age-related mitochondrial changes in rods and cones (Kaarniranta et al., 2020), delaying the normal age-related shrinking of cone inner segments, preserving cone function for longer.

## Data Availability Statement

The original contributions presented in the study are included in the article/Supplementary Material, further inquiries can be directed to the corresponding author/s.

## Ethics Statement

The studies involving human participants were reviewed and approved by the Regional Committee for Medical Research Ethics for the Southern Norway Regional Health Authority. Written informed consent to participate in this study was provided by the participants’ legal guardian/next of kin.

## Author Contributions

RB: conceptualization, formal analysis, supervision, funding acquisition, validation, investigation, visualization, methodology, writing—original draft, project administration, and writing—review and editing. ÅH: data acquisition, formal analysis, investigation, and writing—review and editing. SG: software, data curation, formal analysis, investigation, visualization, writing—original draft, and writing—review and editing. HP: data acquisition and curation, formal analysis, supervision, investigation, visualization, writing—original draft, and writing—review and editing. All authors contributed to the article and approved the submitted version.

## Conflict of Interest

The authors declare that the research was conducted in the absence of any commercial or financial relationships that could be construed as a potential conflict of interest.

## Publisher’s Note

All claims expressed in this article are solely those of the authors and do not necessarily represent those of their affiliated organizations, or those of the publisher, the editors and the reviewers. Any product that may be evaluated in this article, or claim that may be made by its manufacturer, is not guaranteed or endorsed by the publisher.
