# Supplementary material for: The Relationship Between Perifoveal L-Cone Isolating Visual Acuity and Cone Photoreceptor Spacing—Understanding the Transition Between Healthy Aging and Early AMD
Source: Front Aging Neurosci. 2021 Sep 9;13:732287. doi: 10.3389/fnagi.2021.732287 (PMC8458634; doi:10.3389/fnagi.2021.732287)
Supplement: Supplementary file 1 [file Data_Sheet_1.pdf]

Supplementary Material

1 Supplementary Table and Figure

Supplementary Table 1. Qualitative description the OCT images of the macular region of the participants with Early AMD. The AMD severity is classified according to NICE NG82 (2018).

Idnr.	Qualitative description	AMD classification
**5501**	A large vitelliform lesion in the foveal center, and numerous small, medium and large drusen spread throughout the macula. Parafoveal pigment abnormalities.	Early AMD: High risk
**5507**	Medium-large druse with pigment abnormalities in and near the foveal center. No changes observed in the temporal macula.	Early AMD: Medium risk
**5500**	No apparent changes in the foveal center. Pigment abnormalities in the parafoveal area accompanied by disruption of the IZ and EZ nasal inferior for the foveal center.	Early AMD: Medium risk
**5505**	No apparent changes in the foveal center. Numerous small and medium drusen in the macula as well as subretinal drusenoid deposits, especially in the nasal part of the fovea and macula.	Early AMD: Medium risk
**5204**	Small and medium drusen spread within the fovea, parafovea and perifovea.	Early AMD: Low risk
**5504**	Medium drusen in and near the foveal center and inferior to the foveal center and small drusen spread within the para and perifovea.	Early AMD: Low risk
**5506**	No apparent changes in the foveal center. Small and medium drusen in the nasal and inferior part of the macula.	Early AMD: Low risk
**5502**	No apparent changes in or near the foveal center. Medium and small drusen superior and temporal to the fovea.	Early AMD: Low risk
**5503**	A marked thickening of the RPE layer near the foveal center (possibly a low soft druse) and some small drusen (10-15) spread throughout the macula.	Early AMD: Low risk
**5508**	A small druse just temporal of the foveal center. Numerous small and three medium drusen spread within the para and perifovea.	Early AMD: Low risk

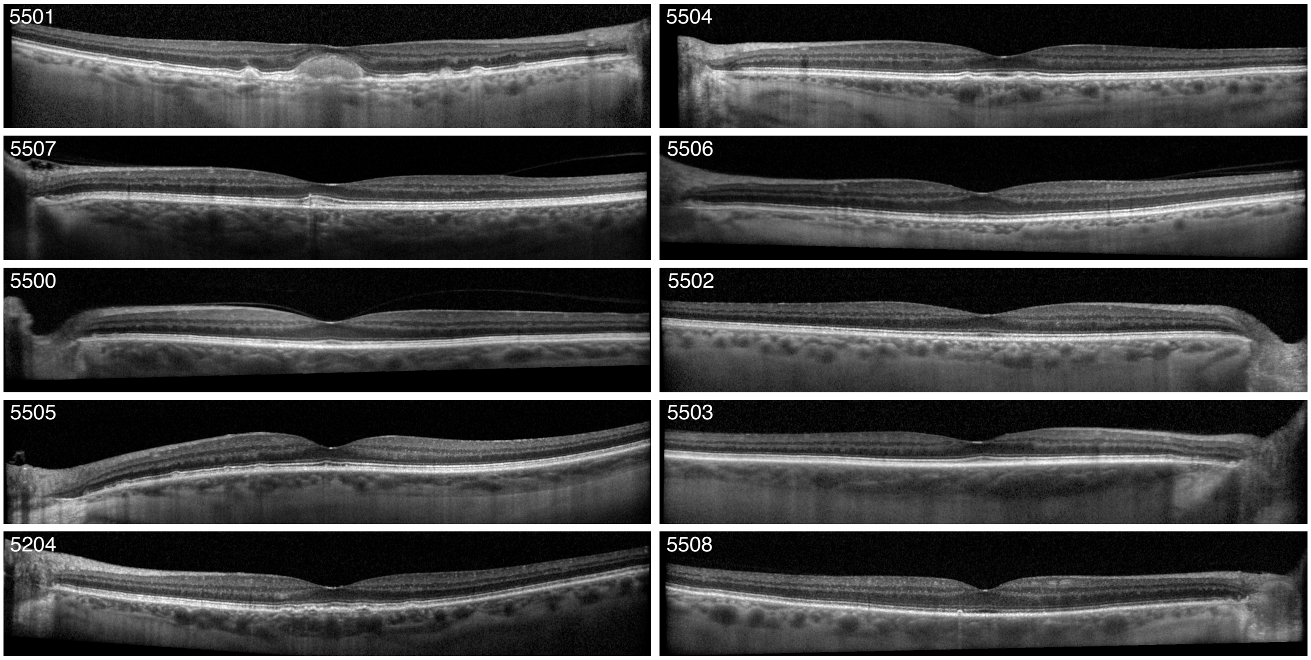

Supplementary Figure 1. Horizonal 30-degrees SD-OCT B-scans through the foveal center of the 10 participants with early AMD sorted according to AMD severity: High risk (5501), medium risk (5507, 5500, 5505) and low risk of progression.

References

NICE guideline [NG82] (2018). *Age-related macular degeneration.* [Online]. https://www.nice.org.uk/guidance/ng82. [Accessed 24 May 2021].
